# Long-term prognostic value of macrophage migration inhibitory factor in ST-segment elevation myocardial infarction patients with metabolic syndrome after percutaneous coronary intervention

**DOI:** 10.3389/fcvm.2022.947395

**Published:** 2022-08-11

**Authors:** Xiao-Lin Yu, Qian Zhao, Fen Liu, Yu-Juan Yuan, Bin-Bin Fang, Xue-He Zhang, Wen-Ling Li, Xiao-Mei Li, Guo-Li Du, Xiao-Ming Gao, Yi-Ning Yang

**Affiliations:** ^1^Department of Cardiology, First Affiliated Hospital of Xinjiang Medical University, Ürümqi, China; ^2^Xinjiang Key Laboratory of Cardiovascular Disease Research, Clinical Medical Research Institute of First Affiliated Hospital of Xinjiang Medical University, Ürümqi, China; ^3^Department of Cardiology, People’s Hospital of Xinjiang Uygur Autonomous Region, Ürümqi, China; ^4^Department of Endocrinology, First Affiliated Hospital of Xinjiang Medical University, Ürümqi, China; ^5^Xinjiang Key Laboratory of Medical Animal Model Research, Ürümqi, China; ^6^State Key Laboratory of Pathogenesis, Prevention and Treatment of High Incidence Diseases in Central Asian, Ürümqi, China

**Keywords:** metabolic syndrome, macrophage migration inhibitory factor, MACCE, ST- segment elevation myocardial infarction, coronary artery disease

## Abstract

Metabolic syndrome (MetS) is a major risk factor for cardiovascular disease and negatively affecting the prognosis of patients with ST elevation myocardial infarction (STEMI). Macrophage migration inhibitory factor (MIF) is a multipotent cytokine involved in various cardiovascular and inflammatory diseases. In this prospective study, we investigate the value of MIF in the long-term prognosis of STEMI combined with MetS after emergency PCI. Circulating MIF levels were measured at admission, and major adverse cardiovascular and cerebrovascular events (MACCE) were monitored during the follow-up period of 4.9 (3.9–5.8) years. MACCE occurred in 92 patients (22.9%), which was significantly higher in MetS (69/255, 27.1%) than in the non-MS subgroup (23/146, 15.8%, *P* < 0.05). Patients with MetS developed MACCE had the highest admission MIF level. Kaplan-Meier survival analysis using the cutoff value of admission MIF (143 ng/ml) showed that patients with a higher MIF level had a greater incidence of MACCE than those with lower MIF levels in both the MetS (*P* < 0.0001) and non-MetS groups (*P* = 0.016). After adjustment for clinical variables, the value of MIF ≥ 143 ng/ml still had the predictive power for the MetS group [HR 9.56, 95% CI (5.397–16.944),*P* < 0.001]; nevertheless, it was not the case in the non-MetS group. Our findings indicated that MetS is a critical risk factor for adverse clinical outcomes in patients with STEMI, and a high admission MIF level has predictive power for the long-term MACCE, which is superior in STEMI patients with MetS and better than other traditional predictors.

## Introduction

Metabolic syndrome (MetS) is characterized by a constellation of metabolic disorders including impaired glucose tolerance, central obesity, dyslipidemia, and hypertension (1). As a result of economic growth and medical advancement, changes in lifestyle and dietary intake and aging have become the major risks for the development of MetS (2–4). The prevalence of MetS has increased significantly worldwide, not only in developed countries but also in developing countries over the past decades. In the United States, the prevalence of MetS was 23.7% (age-adjusted) during 1988–1994 (5), which sharply increased to 32.5–36.9% during 2011–2016 (6). Using the revised National Cholesterol Education Program Adult Treatment Panel III (NCEP ATP III) criteria, the International Collaborative Study of Cardiovascular Disease in ASIA (InterASIA) showed that the prevalence of MetS was 13.7% among adults aged 35–74 years in China in 2001 (3), which increased to 33.9% among adults aged 18 years and older in 2010 based on the China Non-communicable Disease Surveillance data (7). Recently, the China Health and Recruitment Longitudinal Study (CHARLS) revealed a further elevation of MetS prevalence up to 39.7% in middle-aged and elderly Chinese during 2011–2015 (8).

On a global scale, approximately 16.7 million patients die from cardiovascular disease (CVD) every year, representing the leading cause of mortality in the world (9). Growing evidence has strongly indicated that MetS is the major risk factor for CVD (10–14). A meta-analysis including 87 studies involving 951,083 participants reported that MetS was associated with a twofold increased risk of CVD (relative risk, RR: 2.35; 95% CI: 2.02–2.73) and CVD-related mortality (RR: 2.40; 95% CI: 1.87–3.08), and a 1.5-fold elevation in all-cause mortality (RR: 1.58; 95% CI: 1.39–1.78) (15). Thus, patients with MetS were at higher risk for cardiovascular outcomes. Furthermore, MetS was also associated with a higher risk for myocardial infarction (MI) and stroke (15, 16). Patients classified as MetS suffered from acute MI had worse outcomes at follow-up (11). Therefore, early prediction of major adverse cardiovascular and cerebrovascular events (MACCE) in MI patients with MetS bears an important clinical value.

Macrophage migration inhibition factor (MIF) acts as a pro-inflammatory factor and is widely expressed in different cell types and involved in many inflammatory-related disorders (17, 18). Association of MIF in myocardial ischemia and infarction has been reported in clinical and experimental settings (19). Notably, early elevation of plasma MIF levels in patients following ST-segment elevation MI (STEMI) correlated with acute and chronic infarct size and the degree of cardiac remodeling (20). Our previous study demonstrated that admission MIF levels could predict long-term MACCE in patients with STEMI (21). MetS, as a major risk factor for CVD, has drawn great attention in clinical settings. However, it is not known whether admission MIF levels carry the same prognostic importance for development of MACCE in patients with MetS subjected to an acute MI. Furthermore, it is unclear if the same MIF value can be used to predict long-term outcome in both patients with or without MetS after acute MI. The aim of this study is to address these key questions in patients with STEMI after percutaneous coronary intervention (PCI).

## Materials and methods

### Study design and participants

We consecutively recruited patients with STEMI aged >18 years admitted to our hospital from January 2014 to October 2018 who underwent emergency PCI after the onset of chest pain. All patients/participants provided written information. This project is in line with the Declaration of Helsinki, and the research protocol was approved by the Human Ethics Committee of the First Affiliated Hospital of Xinjiang Medical University (Approval ID: K201301-09).

### Inclusion criteria

The diagnosis of STEMI was defined as a plasma level of cardiac high sensitive-troponin T (hs-TnT) >0.1 μg/ml after symptom onset together with at least one of the following: (1) chest pain lasting for >20 min; (2) Electrocardiograph (ECG) exhibiting elevation of ST segment >1 mm or a new pathological Q wave (22). MetS was defined based on the modified NCEP ATPIII criteria (1) and included three or more of the following components: (1) abdominal obesity (body mass index, BMI ≥ 30 for men and ≥ 25 kg/m^2^ for women) (23); (2) elevated triglycerides (TG ≥ 1.69 mmol/L); (3) reduced high-density lipoprotein-cholesterol (HDL-C, < 1.03 mmol/L in men and < 1.29 mmol/L in women); (4) systolic blood pressure (SBP, >130 mmHg) or diastolic blood pressure (DBP >85 mmHg) or use of antihypertensive medications; and (5) fasting plasma glucose >5.6 mmol/L or use of antidiabetic medications. In this study, we used BMI as a surrogate parameter for central obesity, which had been adopted and verified in previous studies (24–27).

### Exclusion criteria

Patients with one or more of the following conditions were excluded: malignancy, thrombolysis, cardiomyopathy, previous history of PCI or coronary artery bypass grafting (CABG), recurrent MI, infectious disease, active inflammatory disease, renal failure, severe liver disease, peripheral arterial disease, or hematologic disease.

### Definition of cardiovascular risk factors

Body mass index was calculated by dividing body weight (kilograms) by the height in meter squares. Overweight/obesity was classified as a BMI ≥ 30 kg/m^2^ for men and ≥ 25 kg/m^2^ for women (27). Persons who reported regular tobacco use in the previous 6 months were considered as current smokers. Hypertension was defined as history of hypertension and/or repeated systemic BP measurements exceeding 140/90 mmHg or use of antihypertensive medications. Diabetes was defined as a history or presence of diabetes and/or a fasting plasma glucose level of >7.0 mmol/L on two separate occasions or a random glucose value >11.1 mmol/L on at least one occasion before the present admission or use of antidiabetic medications. Concentrations of TC >6.2 mmol/L, TG >2.3 mmol/L, LDL-C >4.1 mmol/L, and HDL-C < 1.0 mmol/L were defined as hypercholesterolemia, hypertriglyceridemia, high LDL-C, or low HDL-C, respectively, according to Chinese dyslipidemia guidelines (28). Dyslipidemia was defined as any of the four lipid abnormalities mentioned above. The Global Registry of Acute Coronary Events (Grace) risk score is recognized as a validated predictor of adverse cardiovascular events in patients with AMI (29, 30). It is calculated based on age, heart rate, systolic BP, creatinine level, history of congestive heart failure, PCI and MI, ST-segment changes on admission ECG, and elevated levels of cardiac enzymes or markers.

### Sample collection and laboratory test

Intravenous blood samples were collected at admission for the MIF level test, and the median symptom-to-sampling time was 5.8 h (25th–75th percentile, 3.5–8.0 h). Plasma MIF levels were measured using the Quantikine MIF ELISA kit (R&D Systems, United States) according to the manufacturer’s specifications. The next day after PCI, intravenous blood samples were collected at the Coronary Care Unit and routine whole blood tests and biochemical tests, including high-sensitive C-reactive protein (hs-CRP), were performed in the laboratory of the First Affiliated Hospital Center of Xinjiang Medical University using a commercial automated platform. High sensitive-cardiac troponin T (hs-TnT) was tested at admission and every 4 h after admission to determine the peak.

### Coronary angiography and percutaneous coronary intervention

All patients with STEMI were admitted with aspirin 300 mg, load dose of clopidogrel 300 mg, and standard intravenous heparin 70 U/kg and then underwent emergency coronary angiography followed by PCI. PCI procedures were performed by experienced interventional cardiologists. Multivessel lesions were defined as >50% stenosis in more than one major coronary artery. According to the Gensini score (31, 32), the severity of the injury was 1 (0–25%), 2 (25–50%), 4 (50–75%), 8 (75–90%), 16 (90–99%), and 32 (completely occluded vessels), respectively, and this ratio was multiplied by the segment location weighting factor to obtain the Gensini score for each patient. PCI was considered successful if the patient had a grade 3 blood flow rating for MI thrombolysis (TIMI) in the coronary artery associated with the area of MI and postoperative residual stenosis < 10% (33). After PCI, all patients received dual antiplatelet therapy: 100 mg aspirin, 75 mg clopidogrel daily for at least 1 year, and other cardiovascular-related medications at the discretion of the treating physician.

### Echocardiography

All patients were assessed by transthoracic echocardiography within 48 h after primary PCI using the Vivid 7 Ultrasound System (GE Medical Systems, United States). Standard echocardiography was conducted for the assessment of left ventricular (LV) dysfunction.

### Study endpoints

Study endpoints included MACCE during the follow-up period, including all-cause mortality, target lesion reconstruction, recurrent angina or AMI, readmission due to heart failure, arrhythmia, or/and stroke. All clinical events were defined according to standardized definitions. If patients presented with multiple events, only the first event was considered for event-free survival analysis.

### Follow-up visits

Follow-up visits included telephone interviews, outpatient visits, and inpatient clinical records of readmitted patients. Information on the deceased patient was obtained from hospital records or telephone contacts with relatives of the patient. The follow-up period ended in October 2020. MACCE were recorded at 1, 3, and 6 months after discharge and every 6 months thereafter.

### Quality control

Professionally trained investigators used a uniformly designed questionnaire to collect general patient information, laboratory results, coronary angiography results, and MACCE events through our electronic medical records and paper cases. The database was created using the Epidata 3.0 software, and the data entry was performed by two investigators independently. The data were verified in a batch of 10 again to ensure the accuracy of data entry.

### Statistical analysis

Based on a prospective cohort study design and according to our previous study, the incidence of long-term MACCE in ACS was 38% in the high MIF group, and the hazard ratio was 2.8 (21). We set α = 0.05 and power = 0.9 to calculate the sample size. We also assumed a 10% loss during the follow-up period. Therefore, a sample of 110 patients per group was required. Data were collected using Epidata3.1 (Odense, Denmark) and double checked. Continuous variables with a Gaussian distribution are presented as mean ± standard deviation (SD), and those with a non-Gaussian distribution are presented as median values with corresponding 25th–75th percentiles. The differences between groups were evaluated using Student’s unpaired *t-*test with Welch’s correction or the Mann-Whitney rank test. Categorical variables were expressed as numbers and frequencies, and the difference between groups was detected using the Pearson chi-square test or Fisher exact chi-square test. The cutoff values of admission levels of MIF for predicting MACCE were determined by using the receiver operating characteristic (ROC) plot with the maximal corresponding values of Youden’s index (sensitivity + specificity-1). To visualize the relationship between the cutoff and MACCE during the follow-up, Kaplan-Meier plots were generated, and the log-rank test was used to compare the resulting curves. Potentially influential variables of MACCE among traditional risk factors and variables in univariate cox regression with a value of *P* < 0.05 were tested by collinearity diagnosis in advance, and those variables with interaction between each other and with the variance inflation factor (VIF) ≥ 5 were excluded. The variables that did not show interaction were finally included for a multivariable Cox proportional hazard regression analysis to assess whether a high admission MIF level is an independent predictor of a long-term adverse clinical outcome. Results of univariate and multivariate Cox proportional hazard regression models are presented as a hazard ratio (HR) and 95% confidential interval (CI). *P* < 0.05 was considered statistically significant. Analyses were performed using SPSS Statistics 26 (IBM) and GraphPad Prism 6 (United States).

## Results

### Baseline characteristics of participants

**FIGURE 1:**
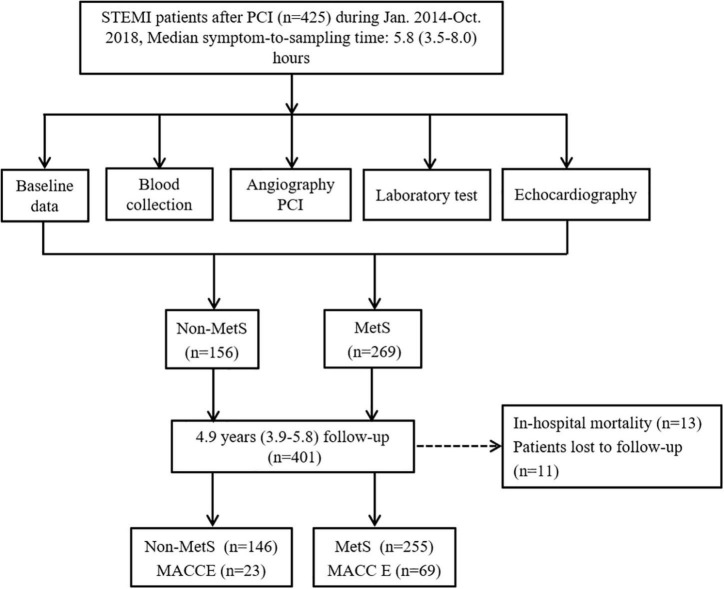
The flowchart of study design with inclusion and exclusion procedures. MACCE, major adverse cardiovascular and cerebrovascular events; PCI, percutaneous coronary intervention; STEMI, ST-elevation myocardial infarction; MS, metabolic syndrome.

The flowchart of the study design is shown in Figure 1. We consecutively recruited 476 patients with STEMI into the study during January 2014–October 2018. Of them, 51 patients were excluded due to thrombolysis therapy, active inflammatory diseases, cancer, renal failure, previous history of MI, and percutaneous transluminal coronary angioplasty (PTCA), and 425 patients with STEMI who received PCI were included. After PCI, patients were divided into non-MetS and MetS groups according to the criteria of MetS diagnosis (1). There were 13 patients who died during hospitalization, and in-hospital mortality was similar between the two groups (4 in non-MetS vs. 9 in MetS, *P* >0.05). After discharge, 412 patients were eligible to enter the follow-up period. We lost contact with 11 (2.6%) patients during the follow-up period. Finally, 401 patients with STEMI were followed up during the 4.9-year period for assessment of long-term clinical outcomes. Baseline characteristics of study participants are presented in Table 1. Although age and gender distribution were similar between the non-MetS and MetS groups, the number of patients in MetS was 1.75-fold more than those in the non-MetS group. It was expected that all variables related to MetS, including percentage of diabetes mellitus and hypertension, and BMI, fasting glucose, total cholesterol (TC), triglycerides (TG), and higher density lipid-cholesterol (HDL-C) were greater in the MetS group (*n* = 255) than in the non-MetS (*n* = 146) group (all *P* < 0.001). White blood cell counting (WBC), hs-CRP, and peak hs-TnT were higher in the MetS group when compared to the non-MetS group (both *P* < *0.05*), indicating more severe systemic inflammation and cardiac injury in the MetS group. Angiographic analysis showed that the MetS group had a higher Gensini score and a prevalence of multivessel disease vs. the non-MetS group (both *P* < 0.05). While the admission MIF level, N-terminal pro-brain natriuretic peptide precursors (NT-proBNP), Grace score, and symptom onset-to-reperfusion time were comparable between the two groups. There was no statistical difference in medication between the non-MetS and MetS groups except for ACEI or ARB drugs (*P* < 0.001) (Table 1).

**TABLE 1:** Baseline clinical characteristics of all participants.

Variables	STEMI (*n* = 401)		*P*-value
	Non-MetS (*n* = 146)	MetS (*n* = 255)	
Age (years)	58.7 ± 11.9	57.2 ± 12.1	*0.222*
Male, *n* (%)	124 (84.9)	204 (80.0)	*0.218*
Current smoker, *n* (%)	82 (56.2)	15 1 (59.2)	*0.551*
Diabetes mellitus, *n* (%)	18 (12.3)	100 (39.2)	<*0.001*
Hypertension, *n* (%)	45 (30.8)	154 (60.4)	<*0.001*
BMI (kg/m^2^)	24.2 ± 3.2	27.5 ± 3.8	<*0.001*
WBC (×10^9^/L)	10.7 ± 3.4	11.4 ± 3.5	*0.033*
Fasting glucose (mmol/L)	8.36 ± 3.59	10.19 ± 4.01	<*0.001*
TC (mmol/L)	5.03 ± 1.12	5.54 ± 1.31	<*0.001*
TG (mmol/L)	1.09 (0.78∼1.59)	2.08 (1.51∼2.91)	<*0.001*
HDL-C (mmol/L)	1.08 ± 0.26	0.88 ± 0.17	<*0.001*
LDL-C (mmol/L)	3.07 ± 0.87	3.04 ± 0.93	*0.759*
NT-proBNP (pg/mL)	342 (80∼994)	422 (116∼1,234)	*0.153*
LVEF (%)	59.2 ± 5.3	59.0 ± 6.2	*0.689*
Peak hs-TnT (ng/mL)	2.02 (0.85∼4.06)	2.50 (1.12∼5.47)	*0.035*
Adm. MIF (ng/ml)	116 ± 55	121 ± 63	*0.368*
hs-CRP (mg/L)	12.2 (4.0∼18.6)	14.1 (6.9∼21.4)	*0.015*
Grace score	154 ± 21	151 ± 24	*0.365*
Gensini score	52 (39∼82)	63 (42∼88)	*0.010*
Multi-vessel disease, *n* (%)	71 (48.6)	155 (60.8)	*0.018*
Symptom onset to reperfusion (h)	5.9 (3.9∼8.4)	6.4 (3.9∼8.6)	*0.612*
Medication at discharge			
Anti-platelet therapy (%)	139 (95.2)	245 (96.1)	*0.676*
ACEIs/ARBs (%)	58 (39.1)	153 (60.0)	<*0.001*
β-blockers (%)	102 (69.9)	166 (65.1)	*0.329*
Statin (%)	142 (97.3)	249 (97.6)	*0.811*

Date are expressed as mean ± SD or median (25th-75th percentiles), or exact number and percentage.

STEMI, ST-segment elevation myocardial infarction; MetS, metabolic syndrome; Adm, admission; MIF, macrophage migration inhibitory factor; hs-CRP, high sensitive C-reactive protein; BMI, body mass index; WBC, white blood cell; TC, total cholesterol; TG, triglyceride; HDL-C, high density lipoprotein-cholesterol; LDL-C, low density lipoprotein-cholesterol; NT-proBNP, N-terminal precursor brain natriuretic peptide; LVEF, left ventricular ejection fraction; hs-TnT, high sensitive-troponin T; Grace, Global Registry of Acute Coronary Events; ACEIs/ARBs, angiotensin converting enzyme inhibitors/angiotensin receptor blocker.

### ST-segment elevation MI patients with metabolic syndrome had a higher incidence of major adverse cardiovascular and cerebrovascular events

During the 4.9 (interquartile range 3.9–5.8) years of follow-up, 92 (22.9%) cases of MACCE were recorded, and the MetS group had a greater occurrence of total MACCE than the non-MetS group (27.1% vs. 15.8%, *P* = 0.010). The category of MACCE is displayed in Table 2. Compared to the non-MetS group, the incidences of all-cause mortality and target lesion revascularization were significantly higher in patients with MetS. The prevalence of other adverse events was comparable between the two groups.

**TABLE 2:** Category of MACCE occurred during the follow-up period.

	Non-MetS (*n* = 146)	MetS (*n* = 255)	*P*-value
Total MACCE, *n* (%)	23 (15.8)	69 (27.1)	*0.010*
All-cause mortality, *n* (%)	1 (0.7)	13 (5.1)	*0.022*
Target lesion revascularization, *n* (%)	2 (1.4)	17 (6.7)	*0.015*
Rehospitalization owing to recurrent angina, *n* (%)	9 (6.2)	18 (7.1)	*0.731*
Rehospitalization owing to AMI, *n* (%)	3 (2.1)	7 (2.8)	*0.753*
Rehospitalization owing to heart failure, *n* (%)	4 (2.7)	8 (3.1)	*1.000*
Rehospitalization owing to Arrhythmia, *n* (%)	3 (2.1)	4 (1.6)	*0.709*
Stroke, *n* (%)	1 (0.7)	2 (0.8)	*1.000*

Date are expressed as exact number and percentage.

MACCE, major adverse cardio- and/or cerebro-vascular events, MetS, metabolic syndrome; AMI, acute myocardial infarction.

### Major adverse cardiovascular and cerebrovascular events and metabolic syndrome associated with higher migration inhibitory factor levels

**FIGURE 2:**
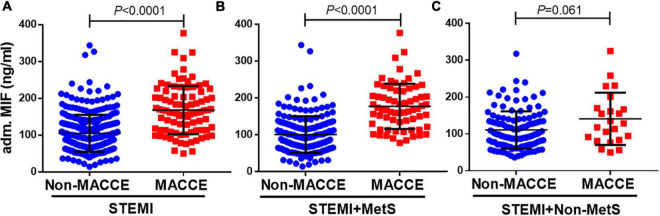
Admission MIF levels between patients with MACCE and non-MACCE. Overall patients with STEMI [**(A)**, non-MACCE, *n* = 309; MACCE, *n* = 92], STEMI + MetS [**(B)**, non-MACCE, *n* = 186; MACCE, *n* = 69], and STEMI + non-MetS [**(C)**, non-MACCE, *n* = 123; MACCE, *n* = 23]. MIF, macrophage migration inhibitory factor.

We further compared admission MIF levels in STEMI patients with or without MACCE, non-MetS STEMI patients with or without MACCE, and MetS STEMI patients with or without MACCE, respectively (Figure 2). The admission MIF level was higher in overall STEMI patients with MACCE vs. non-MACCE patients (165 ± 69 vs. 107 ± 51 ng/ml, *P* < 0.0001, Figure 2A). In STEMI patients with MetS, those who developed MACCE had significantly greater MIF levels than those without MACCE (180 ± 65 vs. 104 ± 50 ng/ml, *P* < 0.0001, Figure 2B). However, in non-MetS STEMI patients, MIF levels were slightly elevated in those with developed MACCE vs. MACCE-free patients but did not reach statistical significance (141 ± 71 vs. 111 ± 51 ng/ml, *P* = 0.061, Figure 2C). As hs-CRP is an inflammatory marker, we also compared its levels in the same way as MIF, and there were no statistical differences between STEMI patients with or without MACCE in overall STEMI patients and STEMI patients with or without MetS (Supplementary Figure 1).

### A higher admission migration inhibitory factor level predicted long-term clinical outcomes and it was superior than other prognostic indicators

**FIGURE 3:**
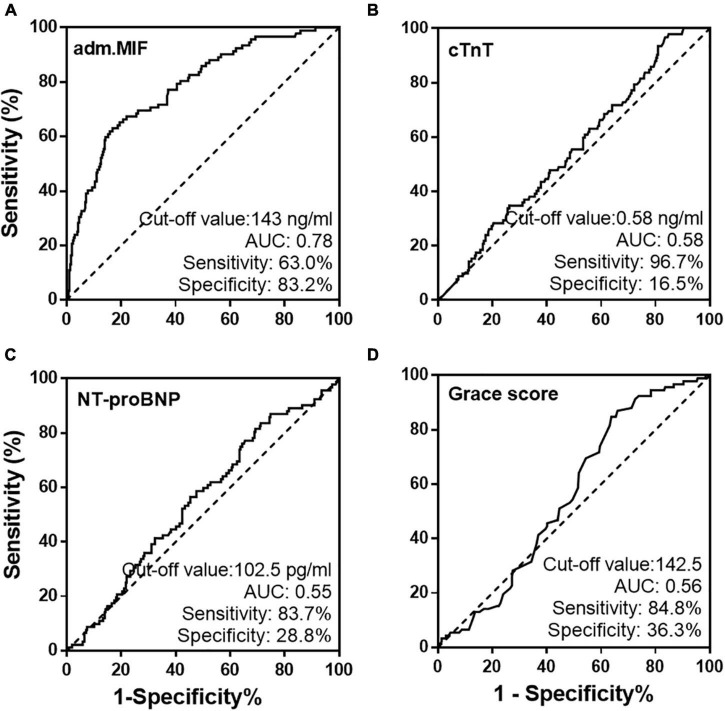
Receiver operating characteristic curves of admission MIF **(A)** and traditional prognostic indicators, cTnT **(B)**, NT-proBNP **(C)**, and Grace score **(D)** from all STEMI patients.

The ROC plots using admission MIF values for all patients with STEMI were generated. The area under the ROC curve for MIF predicting MACCE in patients with STEMI was 0.78 (Figure 3A). The optimal cutoff value for MIF based on the maximum of Youden’s index on the ROC curve was 143 ng/ml with 63.0% sensitivity and 83.2% specificity in predicting long-term clinical outcomes. Using the same method, the cutoff values for hs-TnT, NT-proBNP, and Grace score (Figures 3B–D) and inflammatory indicators (hs-CRP, Supplementary Figure 2A) were calculated, respectively, and the AUCs for those indexes were inferior to the admission MIF.

Based on the cutoff value of admission MIF (143 ng/ml), STEMI patients with or without MetS were further divided into the high- and low-MIF level groups. As shown in Table 3, in the non-MetS group, patients with the high-MIF level (≥ 143 ng/ml) had a greater incidence of diabetes and MACCE than those with the low-MIF level (< 143 ng/ml, both *P* < 0.05). In the MetS group, patients with the high-MIF level had a greater peak level of hs-TnT, Gensini score, and higher incidence of MACCE. Other clinical characteristics, including medications, were similar between the two groups.

**TABLE 3:** Baseline clinical characteristics of STEMI patients with or without MetS grouped by the cut-off value of adm. MIF (143 ng/ml).

Variables	STEMI without MetS (*n* = 146)		*P*-value	STEMI with MetS (*n* = 255)		*P*-value
	Adm. MIF < 143 ng/ml (*n* = 110)	Adm. MIF ≥ 143 ng/ml (*n* = 36)		Adm. MIF < 143 ng/ml (*n* = 179)	Adm. MIF ≥ 143 ng/ml (*n* = 76)	
Age (years)	58.3 ± 11.6	60.1 ± 12.7	*0.445*	57.8 ± 12.1	55.9 ± 12.3	*0.284*
Male	93 (84.5)	31 (86.1)	*0.820*	141 (78.8)	63 (82.9)	*0.451*
Current smoker	64 (58.2)	18 (50.0)	*0.390*	99 (55.3)	52 (68.4)	*0.051*
Diabetes mellitus, *n* (%)	10 (9.1)	8 (22.2)	*0.038*	77 (43.0)	23 (30.3)	*0.056*
Hypertension, n (%)	32 (29.1)	13 (36.1)	*0.428*	106 (59.2)	48 (63.2)	*0.556*
BMI (kg/m^2^)	24.23 ± 3.39	24.09 ± 2.62	*0.814*	27.49 ± 3.82	27.65 ± 3.78	*0.750*
WBC (× 10^9^/L)	10.77 ± 3.37	10.35 ± 3.42	*0.524*	11.39 ± 3.53	11.52 ± 3.56	*0.797*
Fasting glucose (mmol/L)	8.05 ± 2.63	9.31 ± 5.56	*0.199*	10.31 ± 3.91	9.92 ± 4.24	*0.476*
TC (mmol/L)	5.10 ± 1.15	4.83 ± 0.97	*0.205*	5.51 ± 1.33	5.60 ± 1.29	*0.605*
TG (mmol/L)	1.10 (0.78∼1.62)	1.03 (0.78∼1.48)	*0.616*	2.01 (1.45∼2.84)	2.18 (1.74∼3.05)	*0.245*
HDL-C (mmol/L)	1.07 ± 0.28	1.12 ± 0.21	*0.301*	0.88 ± 0.18	0.89 ± 0.17	*0.496*
LDL-C (mmol/L)	3.04 ± 0.87	3.21 ± 0.87	*0.532*	3.05 ± 0.89	3.01 ± 1.02	*0.761*
NT-pro BNP (pg/mL)	342 (79∼894)	383 (80∼1,420)	*0.352*	418 (97∼1,433)	489 (141∼1,157)	*0.906*
LVEF (%)	58.8 ± 5.2	60.3 ± 5.4	*0.132*	58.7 ± 6.6	59.4 ± 4.8	*0.398*
CK-MB max (U/L)	220 (117∼364)	281 (115∼338)	*0.653*	246 (133∼388)	269 (158∼428)	*0.148*
Peak hs-TnT (ng/mL)	1.86 (0.74∼3.95)	2.21 (1.53∼4.20)	*0.150*	2.23 (1.01∼5.31)	3.14 (1.65∼6.90)	*0.013*
hs-CRP (mg/L)	11.3 (3.2∼18.6)	13.4 (8.5∼19.5)	*0.109*	14.1 (6.4∼21.3)	13.4 (7.6∼21.9)	*0.917*
MACCE, *n* (%)	13 (11.8)	10 (27.8)	*0.023*	21 (11.7)	48 (63.2)	<*0.001*
Grace score	154 ± 21	153 ± 23	*0.797*	150 ± 24	153 ± 23	*0.349*
Gensini score	54 (38∼82)	47 (39∼80)	*0.661*	57 (42∼85)	80 (50∼100)	*0.006*
Multi vessel disease, *n* (%)	52 (47.3)	19 (52.8)	*0.566*	106 (59.2)	49 (64.5)	*0.432*
Symptom onset to reperfusion (h)	5.9 (3.9∼8.5)	6.0 (3.6∼8.2)	*0.550*	6.4 (3.9∼9.1)	6.1 (4.1∼8.3)	*0.591*
Medication at discharge						
Anti-platelet therapy (%)	105 (94.6)	34 (97.1)	*1.000*	174 (97.2)	71 (93.4)	*0.169*
ACEIs/ARBs (%)	43 (38.7)	15 (42.9)	*0.664*	102 (57.0)	51 (67.1)	*0.131*
β-blockers (%)	76 (68.5)	26 (74.3)	*0.513*	113 (63.1)	53 (69.7)	*0.311*
Statin (%)	109 (98.2)	33 (94.3)	*0.243*	173 (96.6)	76 (100.0)	*0.183*

Date are expressed as mean ± SD or median (25th–75th percentiles), or exact number and percentage.

STEMI, ST-segment elevation myocardial infarction; MetS, metabolic syndrome; Adm, admission; MIF, macrophage migration inhibitory factor; BMI, body mass index; WBC, white blood cell; TC, total cholesterol; TG, triglyceride; HDL-C, high density lipoprotein-cholesterol; LDL-C, low density lipoprotein-cholesterol; NT-proBNP, N-terminal precursor brain natriuretic peptide; LVEF, left ventricular ejection fraction; hs-TnT, high sensitive-troponin T; hs-CRP, hypersensitive C-reactive protein; Grace, Global Registry of Acute Coronary Events. ACEIs/ARBs, Angiotensin converting enzyme inhibitors/Angiotensin receptor blocker.

**FIGURE 4:**
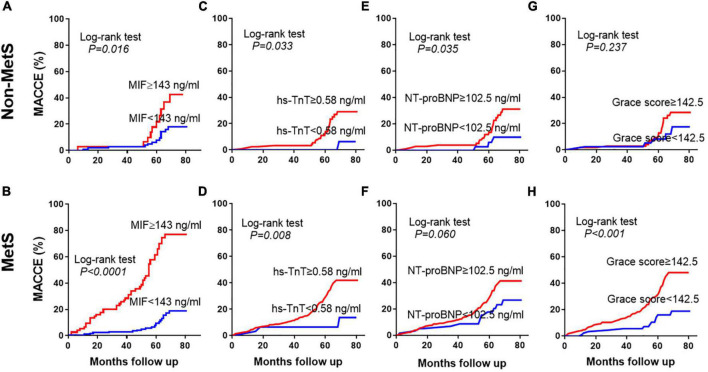
Prediction of MACCE using different prognostic indicators. Kaplan-Meier curves showing the incidence of MACCEs in the non-MetS and MetS groups during the 4.9-year follow-up period using the cutoff values of admission MIF **(A,B)**, hs-TnT **(C,D)**, NT-proBNP **(E,F)**, and Grace score **(G,H)**.

Patients with STEMI discharged from the hospital were separated into two groups using the MIF cutoff value of 143 ng/ml. Cumulative incidences of MACCE using Kaplan-Meier curves are shown in Figure 4. In the STEMI without MetS group, high-MIF level patients had a greater incidence of MACCE (42.4%) than those with low-MIF levels (18.0%, Figure 4A, *P* = 0.016) at the end of the follow-up period. In the STEMI with MetS group, the prevalence of MACCE was markedly higher in patients with high-MIF levels (76.9%) than those with low-MIF levels (18.7%) at the end of a 4.9-year follow-up period (Figure 4B, *P* < 0.001). The predictive power of MIF was much stronger compared to the non-MetS group. The cutoff value (0.58 ng/ml) of hs-TnT had predictive value for MACCE in both the non-MetS and MetS groups (Figures 4C,D, *P* < 0.05). Nevertheless, NT-proBNP with a cutoff value of 102.5 ng/ml predicted MACCE only for the non-MetS group (Figure 4E, *P* = 0.035) but not for the MetS group (Figure 4F, *P* = 0.060). The cutoff value (142.5) of the Grace score had strong predict power for the MetS group (Figure 4H, *P* < 0.001) but not for the non-MetS group (Figure 4G, *P* = 0.237). We also assessed the power of hs-CRP using the cutoff value (17.5 mg/L); it lost the power to predict MACCE in both the non-MetS and MetS groups (Supplementary Figures 2B,C). These results indicate that a higher admission MIF level was associated with a greater incidence of MACCE, which was superior in the MetS group and better than other traditional indicators.

### Elevated admission migration inhibitory factor level is an independent predictor for long-term adverse clinical outcomes

To estimate whether the cutoff value of admission MIF level is an independent factor to predict long-term clinical outcomes, the following statistical methods were applied. First, univariate Cox regression analysis was performed on all participants to screen variables for the next step of multivariate Cox regression analysis, and the results are displayed in Supplementary Table 1. *Second*, a collinearity diagnostic approach was used to exclude potentially interactive variables with VIF ≥ 5, the VIF of all variables presented in Table 3 was less than 5, and therefore, they were eligible for multivariate Cox regression analysis (data not shown). *Third*, based on the nature of square-transformed admission MIF and the cutoff value (≥ 143 ng/ml) of admission MIF generated by the ROC curve, multivariate Cox regression analysis was conducted. Table 4 shows the results of multivariate Cox proportional hazard models used for assessing the independent predictive value of MIF for long-term MACCE. In the crude model, two MIF values had the same predictive capacity for MACCE in both the MetS subgroup and the non-MetS group (*P* < 0.05). However, in Model 1, after adjusting for age, men, history of hypertension and diabetes, and BMI, the predictive power of MIF ≥ 143 ng/ml remained for the MetS group with HR 9.10, 95% CI (5.323–15.568, *P* < 0.001), whereas it was not observed in the non-MetS subgroup. In Model 2, except for those confounding factors used in Model 1, peak TnT, LDL-C, NT-proBNP, LVEF, and Gensini and Grace scores were also included for the adjustment. The value of MIF ≥ 143 ng/ml still had predictive power for the MetS group [HR 9.56, 95% CI (5.397–16.944), *P* < 0.001]. Nevertheless, it was not the case in the non-MetS group. These results demonstrate that a higher admission MIF level (≥ 143 ng/ml) is an independent predictive factor for long-term MACCE in STEMI patients with MetS.

**TABLE 4:** Multivariate cox proportional hazards models for MACCE in both MetS and non-MetS groups.

Group	Variable	Crude model		Model 1		Model 2	
		HR (95% CI)	*P*-value	HR (95% CI)	*P*-value	HR (95% CI)	*P*-value
MetS	MIF level square	1.51 (1.385∼1.655)	<0.001	1.54 (1.407∼1.702)	<0.001	1.60 (1.441∼1.785)	<0.001
	MIF level ≥ 143 ng/ml*	8.17 (4.874∼13.719)	<0.001	9.10 (5.323∼15.568)	<0.001	9.56 (5.397∼16.944)	<0.001
Non-MetS	MIF level square	1.22 (1.034∼1.449)	0.019	1.17 (0.984∼1.397)	0.075	1.23 (1.019∼1.493)	0.031
	MIF level ≥ 143 ng/ml*	2.68 (1.174∼6.115)	0.019	2.41 (0.958∼5.165)	0.101	2.04 (0.805∼6.101)	0.061

Model 1: adjusted for age, male, history of hypertension/diabetes, BMI and admission MIF.

*Model 2: adjusted for model 1 + peak hs-TnT, LDL-C, NT-proBNP, LVEF, Gensini score and Grace score.*

*MACCE, major adverse cardio- and/or cerebro-vascular events; HR, hazard ratio; CI, confidence interval.*

*The cut-off value of 143 ng/ml was generated from the receiver operating characteristics (ROC) curve analysis in all participants.

## Discussion

A number of studies have observed that plasma MIF levels in patients with STEMI were elevated in the early stages after the onset of chest pain (20, 21, 34). Most importantly, the admission MIF level in patients with STEMI was found to be correlated to the size of the myocardial infarction (20), and further, our previous study has shown that admission MIF levels can predict both in-hospital mortality and long-term MACCE (21). These findings demonstrate the potential of admission MIF as a novel biomarker to predict the clinical adverse outcome in the setting of acute MI. MetS is a well-known cardiovascular risk factor, and it has a great negative impact on CVD, especially coronary artery disease (CAD). However, whether admission MIF levels (the earliest available sampling time) also bear a predictive power for the prognosis of patients with STEMI complicated with MetS is unknown.

Our study has made several findings. *First*, the overall MACCE during the 4.9-year (3.9–5.8) follow-up period was 3-fold higher in STEMI patients with MetS compared to those without MetS. This is in line with the negative impact of MetS on CVD (11, 15, 16, 35). The sharply increasing prevalence of MetS, nowadays, has become a global health challenge affecting all nations and races. A similar trend of change in the prevalence of MetS was reported among US adults from 1988 to 2016 (5, 6, 36) and among Chinese adults from 2000 to 2015 (3, 7, 8). Two early studies investigated the relationship between MetS and coronary heart disease (CHD), MI, and stroke, and reported that in men, the MetS age-adjusted relative risk (RR) was 2.54 (95% CI 1.62–3.98) for CHD (10), and the MetS was significantly related in multivariate analysis of MI/stroke with an OR of 2.05 (95% CI 1.64–2.57) (16). A meta-analysis that involved 951,083 participants found that the MetS is associated with a twofold increase in CVD, CVD mortality, MI, and stroke and a 1.5-fold increase in all-cause mortality (15). In an American study, 69% of MetS were detected in 1,129 hospitalized patients due to acute MI, and the worse clinical outcomes (i.e., mortality and rehospitalization) were 38% in patients with MetS vs. 27% in patients with non-MetS during the 12 month follow-up period (11). In the current small-scale study, diagnostic criteria for MetS were met by 63.6% of patients with STEMI during hospitalization and 27.1% of patients with MetS developed MACCE vs. 15.7% in patients with non-MetS during a long-term follow-up period, which is consistent with the results of the American study. These results further support the consensus that MetS is the major cardiovascular risk and clearly raise the alarm that ischemic heart disease concomitant with MetS is more likely to develop adverse clinical outcomes even after blood flow reconstruction. Our findings extend the predictive power of a high admission MIF level from overall patients with STEMI, as we previously reported (21), into the specific high-risk cohort.

*Second*, although the overall admission MIF levels were comparable between non-MetS and MetS groups, they were significantly higher in patients complicated with MetS-developed MACCE than in those MACCE-free counterparts. While the MIF levels in patients without MetS who developed MACCE were also elevated, they did not reach statistical significance when compared MACCE-free counterparts. Furthermore, using the cutoff value of MIF generated by the ROC curve to predict the long-term adverse outcomes, we found that patients in the higher MIF level (≥ 143 ng/ml) subgroup had a greater incidence of MACCE in both the non-MetS and MetS groups during the 4.8-year follow-up period. The predictive power was much higher in patients with MetS. These results demonstrate that a higher admission MIF level was associated with a greater incidence of MACCE, which was superior in the MetS subgroup and better than other traditional prognostic indicators such as hs-TnT, NT-proBNP, and Grace score. Although admission MIF could not differentiate non-MetS and MetS in the very early phase of MI, adverse effects emerged during the follow-up period. This may highlight the negative influence of Mets in this clinical setting.

As demonstrated in our previous study, admission MIF levels from the first available blood samples (as early as 3.5 h after symptom onset) were correlated with myocardial infarct size detected by the golden standard method, magnetic resonance imaging (MRI), at day 3 (the acute phase) and at 3 months (the chronic phase) after acute MI (20). This indicates that the early surge of MIF level, which actually reflects the extent of acute myocardial ischemia/necrosis, likely masks its difference as an inflammatory biomarker between the non-MetS and MetS groups. Supportive evidence of a higher hs-TnT level and greater Gensini score in the high MIF level (≥ 143 ng/ml) subgroup of this cohort signifies more severe myocardial damage. Interestingly, a study using integrated backscatter intravascular ultrasound (IB-IVUS) analyzed coronary plaques and found that the percentage of lipid area and volume was significantly increased, while the percentage of fibrous volume was decreased in stable CAD patients with MetS vs. patients with non-MetS (37). This finding established a direct evidence of MetS is associated with lipid-rich plaque, which contributes to the increasing risk of plaque vulnerability. Moreover, MetS was also found to be associated with a worse no-reflow during emergency PCI for STEMI, and, consequently, a worse prognosis (38). The underlying mechanism is more severe damage to microcirculation (39).

Third, multivariate Cox regression analysis identified that a high admission MIF level is an independent predictor of the long-term MACCE in both the non-MetS and MetS subgroups, which was superior in the MetS subgroup in our study cohort. Therefore, a higher admission MIF level can identify this specific subgroup patients with a higher risk in the setting of acute MI, and it is valuable for cardiologists making a better management in advance. As most risk factors for MetS are modifiable, identification of a high-risk cohort of patients that may benefit from more aggressive risk factor modification (11).

### Study limitations

This current study had several limitations. *First*, the relatively small sample size of this single-center prospective study limited the power of our findings, which requires large-scale multicenter studies to reinforce. *Second*, in our study, only patients with STEMI who received primary PCI were included. The predictive value of admission MIF levels in MetS patients who suffered from acute coronary artery syndrome, especially including STEMI and non-STEMI, and received different interventions such as thrombolysis or CABG should be included in a future study. *Third*, as MetS is systemic inflammatory state (1), if dynamic changes in inflammatory parameters were included in our study, which may better characterize the relationship of the admission MIF level and the prognosis. *Fourth*, infarct size, which is known to influence the final outcomes, was not evaluated. However, we included factors such as peak hs-TnT, Gensini score, percentage of multivessel disease, and the Grace score for further analysis, which may help to minimize this weak point in this current study and validate our findings and conclusion.

## Conclusion

Our study found that STEMI patients with MetS who developed MACCE had significantly higher admission MIF levels than those in MACCE-free patients. STEMI patients with MetS who had an elevated admission MIF level (i.e., ≥ 143 ng/ml) were more likely to develop an adverse clinical outcome after discharge. A higher admission MIF level can be taken as an independent predictive factor to stratify STEMI patients with MetS for a more precise therapy.

## Data availability statement

The original contributions presented in this study are included in the article/Supplementary material, further inquiries can be directed to the corresponding authors.

## Ethics statement

The studies involving human participants were reviewed and approved by the First Affiliated Hospital of Xinjiang Medical University. The patients/participants provided their written informed consent to participate in this study. Written informed consent was obtained from the individual(s) for the publication of any potentially identifiable images or data included in this article.

## Author contributions

X-LY and QZ conducted the study and drafted the manuscript. FL and Y-JY contributed to the statistical analysis. B-BF, W-LL, X-HZ, and G-LD contributed to the data collection and quality control. X-ML, X-MG, and Y-NY were responsible for the funding support and contributed to the study design and revision of the manuscript. All authors contributed to the article and approved the submitted version.

## Abbreviations

MIF: macrophage migration inhibitory factor
STEMI: ST-elevation myocardial infarction
MetS: metabolism syndrome
PCI: percutaneous coronary intervention
CVD: cardiovascular disease
CHD: coronary heart disease
hs-TnT: high sensitive-troponin T
HDL-C: high density lipoprotein cholesterol
LDL-C: low density lipoprotein cholesterol
MACCE: major adverse cardio and cerebrovascular events
WBC: white blood cells
hs-CRP: high sensitive C-reactive protein
Grace score: Global Registry of Acute Coronary Events.

## References

[1] Grundy SM Cleeman JI Daniels SR Donato KA Eckel RH Franklin BA Diagnosis and management of the metabolic syndrome: an American Heart Association/National Heart, Lung, and Blood Institute Scientific Statement. *Circulation.* (2005) 112:2735–52. 10.1161/CIRCULATIONAHA.105.169404 16157765

[2] Lutsey PL Steffen LM Stevens J. Dietary intake and the development of the metabolic syndrome: the Atherosclerosis Risk in Communities study. *Circulation.* (2008) 117:754–61. 10.1161/CIRCULATIONAHA.107.716159 18212291

[3] Gu DF Reynolds K Wu XG Chen J Duan XF Reynolds R Prevalence of the metabolic syndrome and overweight among adults in China. *Lancet.* (2005) 365:1398–405. 10.1016/s0140-6736(05)66375-1 15836888

[4] Xu T Zhu GJ Han SM. Prevalence of and lifestyle factors associated with metabolic syndrome determined using multi-level models in Chinese adults from a cross-sectional survey. *Medicine.* (2020) 99:e22883. 10.1097/MD.0000000000022883 33126337 PMC7598811

[5] Ford ES Giles WH Dietz WH. Prevalence of the metabolic syndrome among US adults: findings from the third National Health and Nutrition Examination Survey. *JAMA.* (2002) 287:356–9. 10.1001/jama.287.3.356 11790215

[6] Hirode G Wong RJ. Trends in the prevalence of metabolic syndrome in the United States, 2011-2016. *JAMA.* (2020) 323:2526–8. 10.1001/jama.2020.4501 32573660 PMC7312413

[7] Lu JL Wang LM Li M Xu Y Jiang Y Wang WQ Metabolic syndrome among adults in China: the 2010 China noncommunicable disease surveillance. *J Clin Endocrinol Metab.* (2017) 102:507–15. 10.1210/jc.2016-2477 27898293

[8] Liu B Chen GQ Zhao RJ Huang D Tao LX. Temporal trends in the prevalence of metabolic syndrome among middle-aged and elderly adults from 2011 to 2015 in China: the China health and retirement longitudinal study (CHARLS). *BMC Public Health.* (2021) 21:1045–55. 10.1186/s12889-021-11042-x 34078325 PMC8173844

[9] Hansson GK. Inflammation, atherosclerosis, and coronary artery disease. *N Engl J Med.* (2005) 352:1685–95. 10.1056/NEJMra043430 15843671

[10] Wilson PWF D’Agostino RB Parise H Sullivan L Meigs JB. Metabolic syndrome as a precursor of cardiovascular disease and type 2 diabetes mellitus. *Circulation.* (2005) 112:3066–72. 10.1161/CIRCULATIONAHA.105.539528 16275870

[11] Arnold SV Lipska KJ Li Y Goyal A Maddox TM McGuire DK The reliability and prognosis of in-hospital diagnosis of metabolic syndrome in the setting of acute myocardial infarction. *J Am Coll Cardiol.* (2013) 62:704–8. 10.1016/j.jacc.2013.02.062 23563136 PMC3765076

[12] Lakka H-M Laaksonen DE Lakka TA Niskanen LK Kumpusalo E Tuomilehto J The metabolic syndrome and total and cardiovascular disease mortality in middle-aged men. *JAMA.* (2002) 288:2709–16. 10.1001/jama.288.21.2709 12460094

[13] Butler J Rodondi N Zhu YW Figaro K Fazio S Vaughan DE Metabolic syndrome and the risk of cardiovascular disease in older adults. *J Am Coll Cardiol.* (2006) 47:1595–602. 10.1016/j.jacc.2005.12.046 16630996

[14] Qin XZ Qiu L Tang GD Tsoi MF Xu T Zhang L Prevalence of metabolic syndrome among ethnic groups in China. *BMC Public Health.* (2020) 20:297–304. 10.1186/s12889-020-8393-6 32143667 PMC7060543

[15] Mottillo S Filion KB Genest J Joseph L Pilote L Poirier P The metabolic syndrome and cardiovascular risk a systematic review and meta-analysis. *J Am Coll Cardiol.* (2010) 56:1113–32. 10.1016/j.jacc.2010.05.034 20863953

[16] Ninomiya JK L’Italien G Criqui MH Whyte JL Gamst A Chen RS. Association of the metabolic syndrome with history of myocardial infarction and stroke in the Third National Health and Nutrition Examination Survey. *Circulation.* (2004) 109:42–6. 10.1161/01.CIR.0000108926.04022.0C 14676144

[17] Morand EF Leech M Bernhagen J. MIF: a new cytokine link between rheumatoid arthritis and atherosclerosis. *Nat Rev Drug Discov.* (2006) 5:399–410. 10.1038/nrd2029 16628200

[18] Yu C-M Lai KW-H Chen YX Huang XR Lan HY. Expression of macrophage migration inhibitory factor in acute ischemic myocardial injury. *J Histochem Cytochem.* (2003) 51:625–31. 10.1177/002215540305100508 12704210

[19] Dayawansa NH Gao XM White DA Dart AM Du XJ. Role of MIF in myocardial ischaemia and infarction: insight from recent clinical and experimental findings. *Clin Sci.* (2014) 127:149–61. 10.1042/CS20130828 24697297

[20] Chan W White DA Wang XY Bai RF Liu Y Yu HY Macrophage migration inhibitory factor for the early prediction of infarct size. *J Am Heart Assoc.* (2013) 2:e000226. 10.1161/JAHA.113.000226 24096574 PMC3835222

[21] Zhao Q Men L Li XM Liu F Shan CF Zhou XR Circulating MIF levels predict clinical outcomes in patients with ST-elevation myocardial infarction after percutaneous coronary intervention. *Can J Cardiol.* (2019) 35:1366–76. 10.1016/j.cjca.2019.04.028 31495686

[22] Jneid H Anderson JL Wright RS Adams CD Bridges CR Casey DE Jr. 2012 ACCF/AHA focused update of the guideline for the management of patients with unstable angina/non-ST-elevation myocardial infarction (updating the 2007 guideline and replacing the 2011 focused update): a report of the American College of Cardiology Foundation/American Heart Association Task Force on Practice Guidelines. *J Am Coll Cardiol.* (2012) 60:645–81. 10.1016/j.jacc.2012.06.004 22809746

[23] Reynolds K Gu DM Whelton PK Wu XG Duan XF Mo JP Prevalence and Risk Factors of Overweight and Obesity in China. *Obesity.* (2017) 15:10–8. 10.1038/oby.2007.527 17228026

[24] Rejas J Bobes J Arango C Aranda P Carmena R Garcia-Garcia M. Concordance of standard and modified NCEP ATP III criteria for identification of metabolic syndrome in outpatients with schizophrenia treated with antipsychotics: a corollary from the CLAMORS study. *Schizophr Res.* (2008) 99:23–8. 10.1016/j.schres.2007.10.015 18063343

[25] Yatskar L Holper E Bansilal S Schwartzbard A Lombardero M Ramanathan K Long-term outcomes in non-diabetic patients with metabolic syndrome undergoing revascularization for multi-vessel coronary artery disease. *Atherosclerosis.* (2008) 198:389–95. 10.1016/j.atherosclerosis.2007.09.046 18061192

[26] Moy FM Bulgiba A. The modified NCEP ATP III criteria maybe better than the IDF criteria in diagnosing Metabolic Syndrome among Malays in Kuala Lumpur. *BMC Public Health.* (2010) 10:678. 10.1186/1471-2458-10-678 21054885 PMC2989964

[27] Zhou JY Liu C Zhou P Li JN Chen RZ Wang Y Prevalence and impact of metabolic syndrome in patients with multivessel coronary artery disease and acute coronary syndrome. *Nutr Metab Cardiovasc Dis.* (2021) 31:2693–9. 10.1016/j.numecd.2021.05.029 34344543

[28] Joint committee for guideline revision. 2016 Chinese guidelines for the management of dyslipidemia in adults. *J Geriatr Cardiol.* (2018) 15:1–29. 10.11909/j.issn.1671-5411.2018.01.011 29434622 PMC5803534

[29] Elbarouni B Goodman SG Yan RT Welsh RC Kornder JM Deyoung JP Validation of the Global Registry of Acute Coronary Event (GRACE) risk score for in-hospital mortality in patients with acute coronary syndrome in Canada. *Am Heart J.* (2009) 158:392–9. 10.1016/j.ahj.2009.06.010 19699862

[30] Shuvy M Beeri G Klein E Cohen T Shlomo N Minha S Accuracy of the Global Registry of Acute Coronary Events (GRACE) risk score in contemporary treatment of patients with acute coronary syndrome. *Can J Cardiol.* (2018) 34:1613–7. 10.1016/j.cjca.2018.09.015 30527149

[31] Gensini GG. A more meaningful scoring system for determining the severity of coronary heart disease. *Am J Cardiol.* (1983) 51:606. 10.1016/s0002-9149(83)80105-2 6823874

[32] Gong P Luo SH Li XL Guo YL Zhu CG Xu RX Relation of ABO blood groups to the severity of coronary atherosclerosis: an Gensini score assessment. *Atherosclerosis.* (2014) 237:748–53. 10.1016/j.atherosclerosis.2014.10.107 25463115

[33] Levine GN Bates ER Blankenship JC Bailey SR Bittl JA Cercek B 2015 ACC/AHA/SCAI focused update on primary percutaneous coronary intervention for patients With ST-elevation myocardial infarction: an update of the 2011 ACCF/AHA/SCAI guideline for percutaneous coronary intervention and the 2013 ACCF/AHA guideline for the management of ST-elevation myocardial infarction. *J Am Coll Cardiol.* (2016) 67:1235–50. 10.1016/j.jacc.2015.10.005 26498666

[34] Yu HY Wang XY Deng XN Zhang YY Gao W. Correlation between plasma macrophage migration inhibitory factor levels and long-term prognosis in patients with acute myocardial infarction complicated with diabetes. *Mediators Inflamm.* (2019) 2019:8276180. 10.1155/2019/8276180 30983881 PMC6431529

[35] Isomaa B Almgren P Tuomi T Forse’n B Lahti K Nisse’n M Cardiovascular morbidity and mortality associated with the metabolic syndrome. *Diabetes Care.* (2001) 24:683–9. 10.2337/diacare.24.4.683 11315831

[36] Aguilar M Bhuket T Torres S Liu B Wong RJ. Prevalence of the metabolic syndrome in the United States, 2003-2012. *JAMA.* (2015) 313:1973–4. 10.1001/jama.2015.4260 25988468

[37] Amano T Matsubara T Uetani T Nanki M Marui N Kato M Impact of metabolic syndrome on tissue characteristics of angiographically mild to moderate coronary lesions integrated backscatter intravascular ultrasound study. *J Am Coll Cardiol.* (2007) 49:1149–56. 10.1016/j.jacc.2006.12.028 17367657

[38] Tartan Z Ozer N Uyarel H Akgul O Gul M Cetin M Metabolic syndrome is a predictor for an ECG sign of no-reflow after primary PCI in patients with acute ST-elevation myocardial infarction. *Nutr Metab Cardiovasc Dis.* (2008) 18:441–7. 10.1016/j.numecd.2007.02.015 17981019

[39] Uchida Y Ichimiya S Ishii H Kanashiro M Watanabe J Yoshikawa D Impact of metabolic syndrome on various aspects of microcirculation and major adverse cardiac events in patients with ST-segment elevation myocardial infarction. *Circ J.* (2012) 76:1972–9. 10.1253/circj.cj-11-1299 22664935

