# Supplementary material for: Long-term prognostic value of macrophage migration inhibitory factor in ST-segment elevation myocardial infarction patients with metabolic syndrome after percutaneous coronary intervention
Source: Front Cardiovasc Med. 2022 Aug 11;9:947395. doi: 10.3389/fcvm.2022.947395 (PMC9403533; doi:10.3389/fcvm.2022.947395)
Supplement: Supplementary file 1 [file Data_Sheet_1.pdf]

Manuscript ID: 947395R2

Long-term prognostic value of macrophage migration inhibitory factor in ST-segment elevation myocardial infarction patients with metabolic syndrome after percutaneous coronary intervention

Xiao-LinYu[1]†, Qian Zhao[1.2]†, Fen Liu[1,2], Yu-Juan Yuan[3], Bin-Bin Fang[2], Xue-He Zhang[1.2], Wen-Ling Li[1.2], Xiao-Mei Li[1,2], Guo-Li Du[4,5], Xiao-Ming Gao[2,5,6]* and Yi-Ning Yang[1,2,3,6]*

[1]Department of Cardiology, The First Affiliated Hospital of Xinjiang Medical University, Urumqi, China,
[2]Xinjiang Key Laboratory of Cardiovascular Disease Research, Clinical Medical Research Institute of First Affiliated Hospital of Xinjiang Medical University, Urumqi, China,
[3]Department of Cardiology, People's Hospital of Xinjiang Uygur Autonomous Region, Urumqi, China,
[4]Department of Endocrinology, The First Affiliated Hospital of Xinjiang Medical University, Urumqi, China,
[5] Xinjiang Key Laboratory of Medical Animal Model Research, Urumqi, China
[6] State Key Laboratory of Pathogenesis, Prevention and Treatment of High Incidence Diseases in Central Asian, Urumqi, China

Supplementary materials

Supplement Table 1. Univariate Cox regression analysis for the traditional predictors of MACCE.

Risk Factors	Overall STEMI			
	HR	95% CI		*P*
Age (yrs)	1.023	1.005	1.041	0.011
Male	0.690	0.424	1.123	0.135
Current smoker	1.178	0.782	1.775	0.433
Diabetes mellitus	1.108	0.711	1.728	0.650
Hypertension	1.322	0.877	1.992	0.183
BMI (kg/m^2)	1.003	0.955	1.054	0.891
WBC ($\times 10^9$/L)	1.004	0.947	1.064	0.895
TC (mmol/L)	1.018	0.865	1.198	0.832
TG (mmol/L)	1.011	0.891	1.147	0.868
HDL-C (mmol/L)	0.530	0.207	1.358	0.186
LDL-C (mmol/L)	1.028	0.816	1.294	0.817
NT-proBNP (pg/mL)	1.000	1.000	1.000	0.422
hs-CRP (mg/L)	1.003	0.995	1.011	0.438
LVEF (%)	0.983	0.952	1.015	0.302
Peak hs-TnT (ng/mL)	1.089	1.021	1.163	0.010
Adm. MIF (ng/ml)	1.015	1.012	1.018	<0.001
Grace score	1.009	1.001	1.018	0.035
Gensini score	1.008	1.003	1.014	0.001
Multi-vessel disease	1.011	0.671	1.524	0.958
Symptom onset to reperfusion (h)	0.964	0.915	1.016	0.176

MACCE, major adverse cardio- and/or cerebro-vascular events; STEMI, ST-segment elevation myocardial infarction; HR, hazard ratio; CI, confidence interval; MetS, metabolic syndrome; Adm, admission; MIF, macrophage migration inhibitory factor; BMI, body mass index; WBC, white blood cell; TC, total cholesterol; TG, triglyceride; HDL-C, high density lipoprotein-cholesterol; LDL-C, low density lipoprotein-cholesterol; NT-proBNP, N-terminal precursor brain natriuretic peptide; hs-CRP, high sensitive C-reactive protein; LVEF, left ventricular ejection fraction; hs-TnT, high sensitive-troponin T; Grace, Global Registry of Acute Coronary Events.

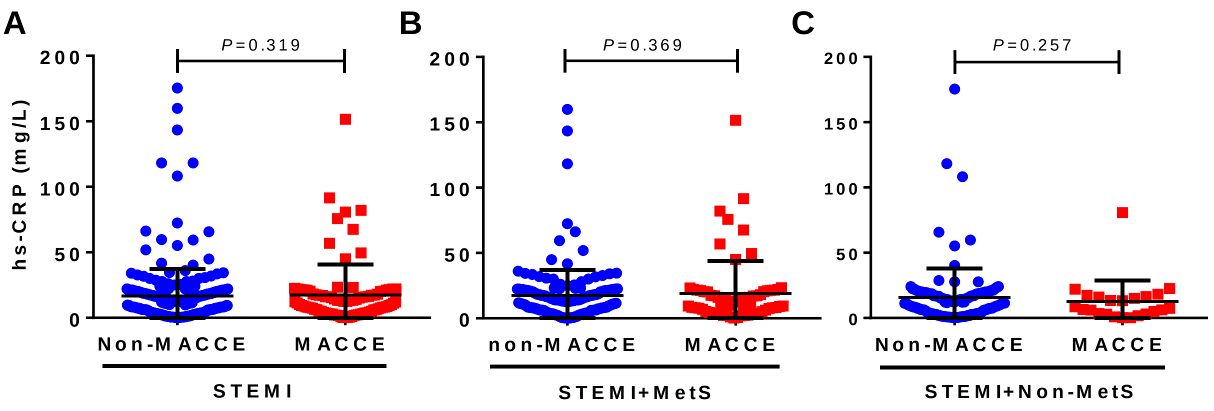

Supplement Figure 1: High sensitive C-reactive protein (hs-CRP) between patients developed MACCE and non-MACCE. Overall STEMI patients (A, non-MACCE, n=309; MACCE, n=92), STEMI+ MetS (B, non-MACCE, n=186; MACCE, n=69) and STEMI+ non-MetS (C, non-MACCE, n=123; MACCE, n=23).

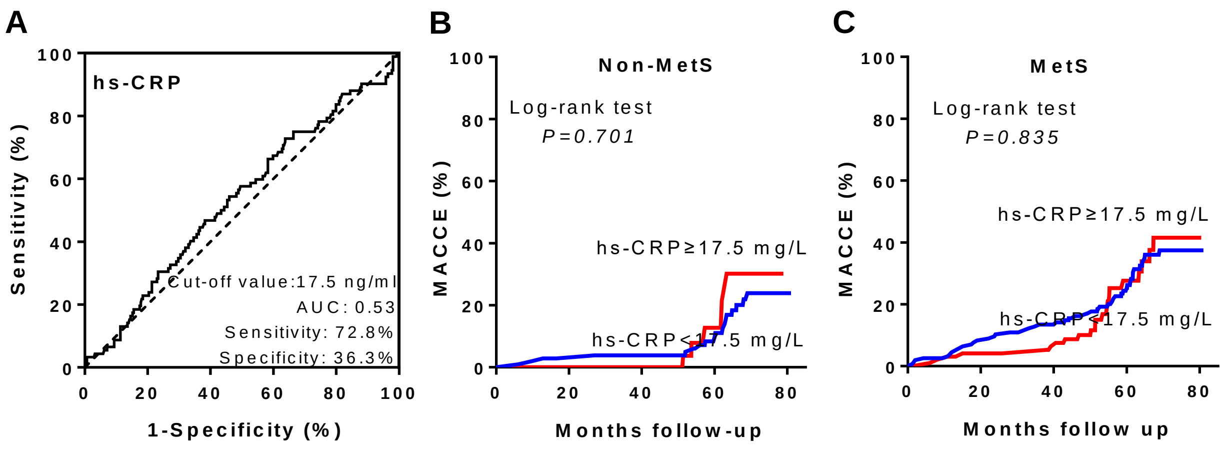

Supplement Figure 2: Receiver operating characteristic curves (A) and Kaplan-Meier curves showing the incidence of MACCEs in non-MetS (B) and MetS group (C) during the 4.9-year (3.9-5.8) follow-up period.
